# Genome-Wide Discovery of Long Non-Coding RNAs in Rainbow Trout

**DOI:** 10.1371/journal.pone.0148940

**Published:** 2016-02-19

**Authors:** Rafet Al-Tobasei, Bam Paneru, Mohamed Salem

**Affiliations:** 1 Computational Science Program, Middle Tennessee State University, Murfreesboro, TN, 37132, United States of America; 2 Department of Biology and Molecular Biosciences Program, Middle Tennessee State University, Murfreesboro, TN, 37132, United States of America; INRA, FRANCE

## Abstract

The ENCODE project revealed that ~70% of the human genome is transcribed. While only 1–2% of the RNAs encode for proteins, the rest are non-coding RNAs. Long non-coding RNAs (lncRNAs) form a diverse class of non-coding RNAs that are longer than 200nt. Emerging evidence indicates that lncRNAs play critical roles in various cellular processes including regulation of gene expression. LncRNAs show low levels of gene expression and sequence conservation, which make their computational identification in genomes difficult. In this study, more than two billion Illumina sequence reads were mapped to the genome reference using the TopHat and Cufflinks software. Transcripts shorter than 200nt, with more than 83–100 amino acids ORF, or with significant homologies to the NCBI nr-protein database were removed. In addition, a computational pipeline was used to filter the remaining transcripts based on a protein-coding-score test. Depending on the filtering stringency conditions, between 31,195 and 54,503 lncRNAs were identified, with only 421 matching known lncRNAs in other species. A digital gene expression atlas revealed 2,935 tissue-specific and 3,269 ubiquitously-expressed lncRNAs. This study annotates the lncRNA rainbow trout genome and provides a valuable resource for functional genomics research in salmonids.

## Introduction

Global gene expression data in different mammalian species have demonstrated that protein-coding sequences occupy less than 2% of the genome, and the vast majority of the genome is transcribed into non-coding RNAs [1–4]. These non-coding RNA molecules include small nuclear RNA (snRNA), small nucleolar RNA (snoRNA), microRNA (miRNA), small interfering RNA (siRNA), piwi RNA (piRNA), signal recognition particle (SRP) RNA and lncRNA. LncRNAs are defined as non-protein-coding RNAs greater than 200 nucleotides in length, distinguishing them from small non-coding RNAs [5, 6]. Based on their proximity to the protein-coding genes in a genome, lncRNAs are subdivided as genic (intronic or exonic with sense, antisense, and bidirectional orientation) or intergenic [7, 8]. Unlike small non-coding RNAs, lncRNA sequences are less conserved and are expressed at relatively low levels, and these characteristics make their computational identification and annotation difficult [9].

Like protein-coding genes, lncRNAs are often transcribed by RNA polymerase II and can be post transcriptionally modified by splicing, capping and polyadenylation [10–13]. In contrast to protein-coding genes, a majority of lncRNA transcripts tend to have fewer exons [9] and a shorter transcript size (average of 800 nucleotides) [14]. LncRNAs usually exhibit highly cell- and tissue-specific expression patterns and sometimes they are uniquely localized to a specific cellular compartment [15–18].

Even though a small number of lncRNAs have experimentally validated molecular functions, a substantial number of lncRNAs have been functionally annotated. LncRNAs are considered important gene regulators due to, at least, three important molecular roles; these RNAs serve as decoys, scaffolds or guides. Many lncRNAs serve as decoys that preclude access to DNA by regulatory proteins; this role affects transcription of protein-coding genes [19, 20]. Some lncRNAs regulate genes by acting as scaffolds to bring two or more proteins into a discrete complex [21–24]. Other lncRNAs regulate different developmental and cellular processes by guiding a specific protein complexes to a specific promoter in response to certain molecular signals [25–27]. LncRNA mediated guidance of chromatin modifying proteins affects expression of neighboring genes (*cis*) or distant genes (*trans*) and there is evidence that even cis acting lncRNAs have ability to act in *trans* [28–30]. Beside transcriptional control, lncRNAs regulate many molecular processes including alternative splicing [31, 32], other post transcriptional processes [33], and mRNA transport [34].

Aquaculture of rainbow trout supplies a significant portion of aquatic food in the USA and worldwide. In addition to its importance as a food species, rainbow trout is one of the most widely used fish species as a model in biomedical research [35–42]. In order to improve aquaculture production and efficiency and facilitate biomedical research of involving rainbow trout, a great deal of genetic information has been accumulated for this species that includes a recently published initial draft of the genome [4] and several assemblies of the transcriptome [43–45]. However, a complete understanding of the trout’s genome biology is still lacking. Recent studies in mammalian and non-mammalian species have resolved some long-standing mysteries in biology by functionally characterizing lncRNAs as important regulators of protein-coding genes [24, 46–50]. With growing interest in lncRNAs-mediated gene regulation, these RNAs have been characterized, genome-wide, in limited animal and plant species in recent years [15, 51]. And, our knowledge of lncRNAs in fish is still very limited [52]. Therefore, the objective of this study was to identify and characterize lncRNAs in rainbow trout genome and create a global gene expression atlas of lncRNAs in several vital tissues.

## Materials and Methods

### Ethics Statement

Institutional Animal Care and Use Committee of The United States Department of Agriculture, National Center for Cool and Cold Water Aquaculture (Leetown, WV) specifically reviewed and approved all husbandry practices used in this study (IACUC approval #056).

### Data Source

To facilitate lncRNA discovery in rainbow trout, four high-throughput sequence datasets were used in this study. 1) About 1.16 billion Illumina sequence reads as we previously described [43]. Briefly, 13 tissues including brain, white muscle, red muscle, fat, gill, head kidney, kidney, intestine, skin, spleen, stomach, liver and testis were sequenced from a single male-doubled haploid rainbow trout. Sequencing libraries were constructed using poly-A selection technique and cDNA libraries were sequenced using Illumina’s paired-end protocol. Data were generated from a single doubled haploid individual to overcome the assembly bioinformatics challenges of the trout duplicated genome. 2) Similarly, about 0.75 billion Illumina single reads, used in annotating the rainbow trout genome and sequenced from a doubled haploid female rainbow trout, as previously described by Berthelot et al.[53]. Briefly, 13 vital tissues including (liver, brain, heart, skin, ovary, white and red muscle, anterior and posterior kidney, pituitary gland, stomach, gills) were sequenced. Sequencing libraries were constructed using poly-A selection technique and cDNA libraries were sequenced using Illumina’s 101 base-lengths single read protocol. 3) About 0.25 billion reads used in assembling the anadromous steelhead (Oncorhynchus mykiss) transcriptome by Fox et al. [45]. 4) About 89 million reads data set from redband trout (Oncorhynchus mykiss) by Narum *et al*. [54]. Data from Narum *et al*. were chosen because Ribo-Zero RNA-Seq libraries were sequenced to capture both the polyadenylated and the non- polyadenylated RNAs with information about transcript strand orientation.

### Computational Prediction Pipeline

Sequencing reads were mapped to the genome reference [4] using the TopHat and Cufflinks software packages [55]. An in house Perl script was written to filter the transcripts shorter than 200 nt. Several stages of filtration were performed to remove protein-coding transcripts and small non-coding RNAs. First, transcripts were searched against NCBI nr protein database (updated on 10/01/2014). All the transcripts which had an open reading frame more than 100 amino acids were removed. Next, protein-coding calculator (CPC) was used to remove any remaining potential protein-coding transcripts (Index value <-0.5) [56]. To remove other classes of RNAs (tRNA, rRNA, snoRNA, miRNA, siRNA and other small non-coding RNAs) transcripts were searched against multiple RNA databases including genomic tRNA database, mirBase, LSU (large subunit ribosomal RNA) and SSU (Small subunit ribosomal RNA) databases [57–60]. Any transcripts which showed sequence similarity with any of these classes of RNAs with cut-off E value of ≤ 0.0001 were removed. After these filtration steps, putative lncRNA transcripts were searched against several noncoding-RNA databases to explore sequence similarity of putative rainbow trout lncRNAs transcripts to previously characterized lncRNAs in other species [52, 61–65]. All prediction steps were applied independently to the four transcriptome datasets. All putative lncRNAs from all four datasets were blasted against each other. LncRNA which were identified in at least 2 of the 4 datasets were chosen for further analysis. Data set from Narum et al., is the only one with information about strand orientation [54]. To ensure correct sense and antisense orientations of lncRNAs from the other three sources, their strand orientation was assigned by matching to counterparts from Narum and coworkers (based on sequence similarity match of more than 95% and same genomic location coordinates). A total of 54,503 non-redundant lncRNAs were identified in this dataset.

For the extra filtration steps, more stringently selected lncRNAs, any putative lncRNA containing ORF covering more 35% of its length or more than 83 amino acid were filtered out [66]. In addition, the cut-off value for the CPC [56] was decreased from -0.5 to -1.0. Further, if any lncRNA overlapped with more than 100 nt with another lncRNA from a different dataset, we filtered out the shortest lncRNA. Furthermore, any lncRNA that overlapped with a protein-coding gene in the sense orientation was removed. Lastly, any single-exon lncRNA that was adjacent to a protein-coding gene within 500nt was removed.

### Identification of Tissue Expression

For lncRNA tissue distribution, sequencing reads from 13 tissues were independently mapped to all putative lncRNAs and gene expression level were measured in terms of RPKM. House-keeping and tissue-specific genes were determined as we previously described [43].

### Gene Clustering

Sequencing reads from each tissue were mapped to combined reference consisting of the lncRNAs and mRNAs from the rainbow trout genome reference [4]. Expression of lncRNAs and protein-coding genes was determined in terms of RPKM. Expression value of each transcripts in each tissue was normalized using a scaling method in CLC genomics workbench with mean as the normalization value. Normalized expression values of transcripts in each of the 13 studied tissues were used to cluster protein-coding genes and lncRNAs using a clustering feature in Multi-experiment Viewer (MeV) program [67, 68]. The minimum correlation threshold to generate clusters was 0.97.

### Identification of Genomic Location of lncRNAs Relative to Neighboring Protein-Coding Genes

LncRNAs were classified based on their intersection or relative location to protein-coding genes using in-house Perl scripts using the rainbow trout genome data (downloaded from http://www.genoscope.cns.fr/trout/data/).

## Results and Discussion

### Identification of Putative lncRNAs in Rainbow Trout

**Fig 1:**
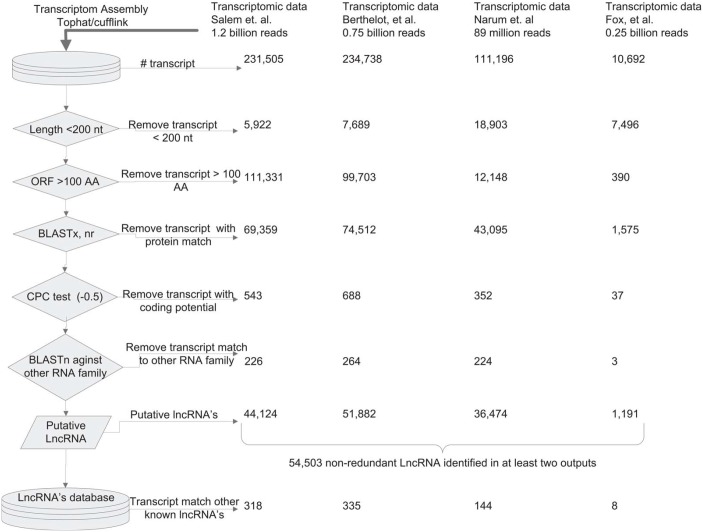
Bioinformatics pipeline used in prediction of rainbow trout lncRNAs. LncRNAs were predicted from four different transcriptomic datasets, then all putative lncRNAs from all data were blasted against each other. A total of 54,503 non-redundant lncRNAs identified in at least 2 of the 4 data sets were chosen for further analyses in order to increase the confidence of lncRNA prediction. Vertical arrows are pointing toward the subsequent prediction and filtration steps of the workflow. First horizontal arrow pointing toward the right is referring to the number of initial transcripts predicted from the four datasets. Middle six horizontal arrows indicate the number of transcripts filtered at each step and the final horizontal arrow points to the number of putative lncRNAs with significant hits to noncoding-RNA databases from each dataset.

The main objective of this study was to identify a comprehensive list of putative lncRNA genes in the rainbow trout genome. To accomplish this, we sequenced poly-A selected cDNA libraries using total RNA isolated from 13 tissues. Recently, we used the same sequencing data to identify protein-coding transcripts in the trout genome [43]. In this study, sequence data for about 1.167 billion, paired-end reads (100 nt) were mapped against a reference rainbow trout genome using the Cufflink and TopHat software [55, 69], resulting in 231,505 putative transcripts. Several filtration steps were used to distinguish lncRNAs in the transcript list by removing the protein-coding transcripts, pseudogenes and other classes of non-coding RNAs including rRNA, miRNA, tRNA, snRNA, snoRNA (Fig 1). First, all transcripts shorter than 200 nt were removed, and then transcripts with an open reading frame (ORF) longer than 100 amino acids were filtered out. Next, remaining transcripts were BLASTx searched against the NCBI non-redundant protein database to eliminate transcripts with sequence similarity to known proteins at a cut off E-value of ≤ 0.0001. To further filter remaining protein-coding transcripts, we used the Coding Potential Calculator (CPC) software that assesses quality and completeness of query ORF to proteins in the NCBI database using six biologically meaningful sequence features [56]. These filtration steps left 44,350 transcripts from this data set that had very little or no evidence of protein-coding ability. Because most of the small non-coding RNAs like miRNA and tRNA are shorter than 200 nt, the first filtration step should be enough to remove most of the small non-coding RNAs. To confirm removal of any remaining small non-coding RNAs (tRNA, rRNA, snoRNA, miRNA, siRNA and other small non-coding RNAs), transcripts were searched against multiple RNA databases including genomic tRNA database, mirBase, and LSU (large subunit ribosomal RNA) and SSU (Small subunit ribosomal RNA) databases [57–60]. After application of the above filtration steps, we found 44,124 putative lncRNAs from our sequence data set (Salem et al., [43]). These lncRNAs exhibited little or no evidence of coding potential or belonging to other non-coding classes of RNA.

Because some of the lncRNAs are thought to be due to expression noise [70], we conceptualized that prediction of lncRNAs from different reliable data sources would be an important step in removing false lncRNAs. To achieve this goal, the same lncRNAs prediction pipeline was applied to discover putative lncRNAs from three other rainbow trout transcriptomic datasets that are available on NCBI (Fig 1). Those three sources were sequence data used by Berthelot et al. [4] in annotating the rainbow trout genome, a data set used by Fox et al. [45] in assembling the anadromous steelhead (*Oncorhynchus mykiss*) transcriptome and a data set from redband trout (*Oncorhynchus mykiss*) that was reported by Narum et al. [54]. Data from Narum et al. were particularly useful because Ribo-Zero RNA-Seq protocols were used which allow sequencing both the polyadenylated and the non- polyadenylated RNAs. In addition, the strand orientation sequence information was preserved. From these three sequence data sources, a total of 0.75B reads, 89M reads, and 0.25B reads were used in the prediction pipeline that yielded 51,882; 1,191; and 36,474 putative lncRNAs in the three datasets, respectively. LncRNAs predicted in at least 2 of the 4 data sets were considered for the subsequent analyses. After removal of redundant transcripts, we had a total of 54,503 putative lncRNAs. Fig 1 illustrates the bioinformatics pipeline used in prediction of lncRNAs in all four datasets, and Table 1 and S1 table report the number of putative lncRNAs predicted in each dataset. FASTA and gtf annotation files are available at http://www.animalgenome.org/repository/pub/MTSU2015.1014/.

**Table 1:** Number of lncRNA predicted in at least 2 of the 4 datasets and final numbers after merging and removal of redundant sequences.

Source	LncRNAs common between two data sources				Putative non-redundant lncRNA from each sources after combining all four sources	
	Salem et. al.	Berthelot et. al.	Narum et. al.	Fox et. al.	Source	Number
Salem et. al.	**x**	35,307	13,557	268	Salem et. al.	21,617
Berthelot et. al.	35,307	**x**	13,993	291	Berthelot et al.	22,568
Narum et. al	13,557	13,993	**x**	401	Narum et. al	10,097
Fox et. al.	268	291	401	**x**	Fox et al.	221
					**Total**	**54,503**

To look for evolutionarily conserved lncRNAs in rainbow trout, all putative lncRNA transcripts (54,503) were searched against several noncoding-RNA databases (E ≤ 0.0001) [52, 61–65]. Of those 54,503 lncRNAs, only 421 had sequence homology to lncRNAs from other species (S1 table). This low evolutionary conservation of lncRNAs is in agreement with previous reports [9].

### Characterization of lncRNAs

**Fig 2:**
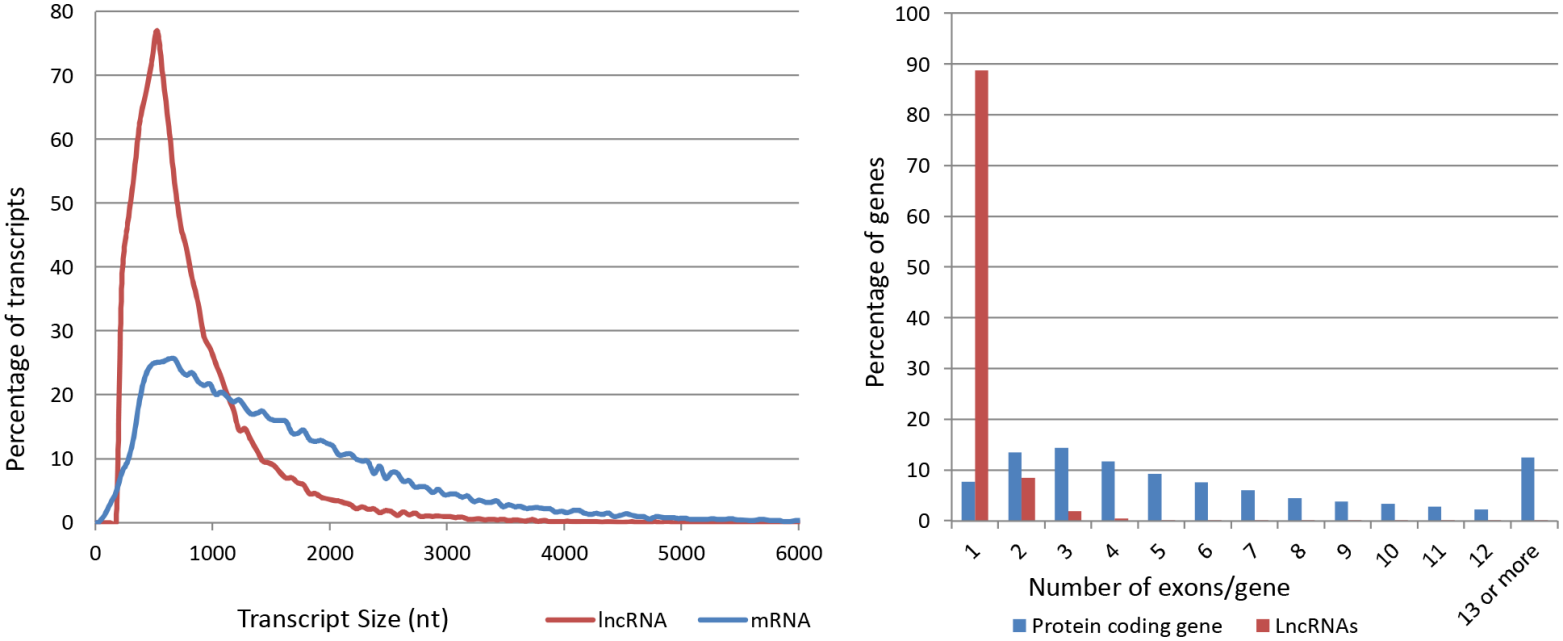
Distribution of sequence length of LncRNAs compared to protein-coding transcripts in rainbow trout. LncRNAs were shorter than protein-coding genes with (0.821 kb) and (1.636 kb) average length in each, respectively (Left). Distribution of number of exons in LncRNAs compared to that of protein-coding genes. Most LncRNA transcripts (~90%) have only one exon whereas majority of the protein-coding transcripts tend to have two or more exons (Right).

Studies on mouse, zebra fish and maize have suggested that lncRNAs are shorter than protein-coding genes, have relatively fewer exons, and are expressed at a lower level [51, 52, 71]. Consistent with previous reports, our study indicates that trout lncRNAs were shorter (0.821 kb) than protein-coding genes (1.636 kb) (Fig 2). In addition, the average number of exons in lncRNAs was 1.14 compared to 4.75 in protein-coding genes. Unlike the trout protein-coding genes, ~90% of the trout lncRNAs had one exon. Fig 2 and Table 2 show distribution and number of exons in lncRNAs compared to protein-coding genes. Data regarding exon numbers in lncRNAs from different species are inconsistent. Similar to our findings, some plant and animal studies reported one-exon bias for lncRNAs [51, 72]. Conversely, some human studies showed a remarkable two-exon prevalence in the majority of lncRNAs [9]. Several reasons may explain these discrepancies including tissue variation, developmental stages, sequencing techniques and biases due to variations in number and length of genes in different species.

**Table 2:** Number of exons and average length of lncRNAs in different data sets.

	Salem et al.		Berthelot et al.		Narum et al.		Fox et al.		Common	
# of exon	LncRNA %	Average length	LncRNA %	Average length	LncRNA %	Average length	LncRNA %	Average length	LncRNA %	Average length
1	86.14	790	88.52	682	96.62	453	98.24	353	88.84	796
2	10.63	888	8.71	846	2.79	462	1.34	377	8.49	1007
3	2.37	973	2.07	893	0.43	480	0.42	359	1.91	1044
4	0.51	1090	0.47	1030	0.1	475	0	0	0.46	1225
5	0.15	1284	0.11	1217	0.02	792	0	0	0.13	1390
6	0.08	1289	0.04	1157	0.02	514	0	0	0.07	1206
7	0.05	1379	0.03	1076	0.01	477	0	0	0.03	1183
8	0.03	1322	0.01	1227	0	631	0	0	0.02	1364
9	0.01	1217	0.01	1394	0.01	620	0	0	0.01	1302
10	0.02	1167	0.01	1199	0	0	0	0	0.01	1181

**Fig 3:**
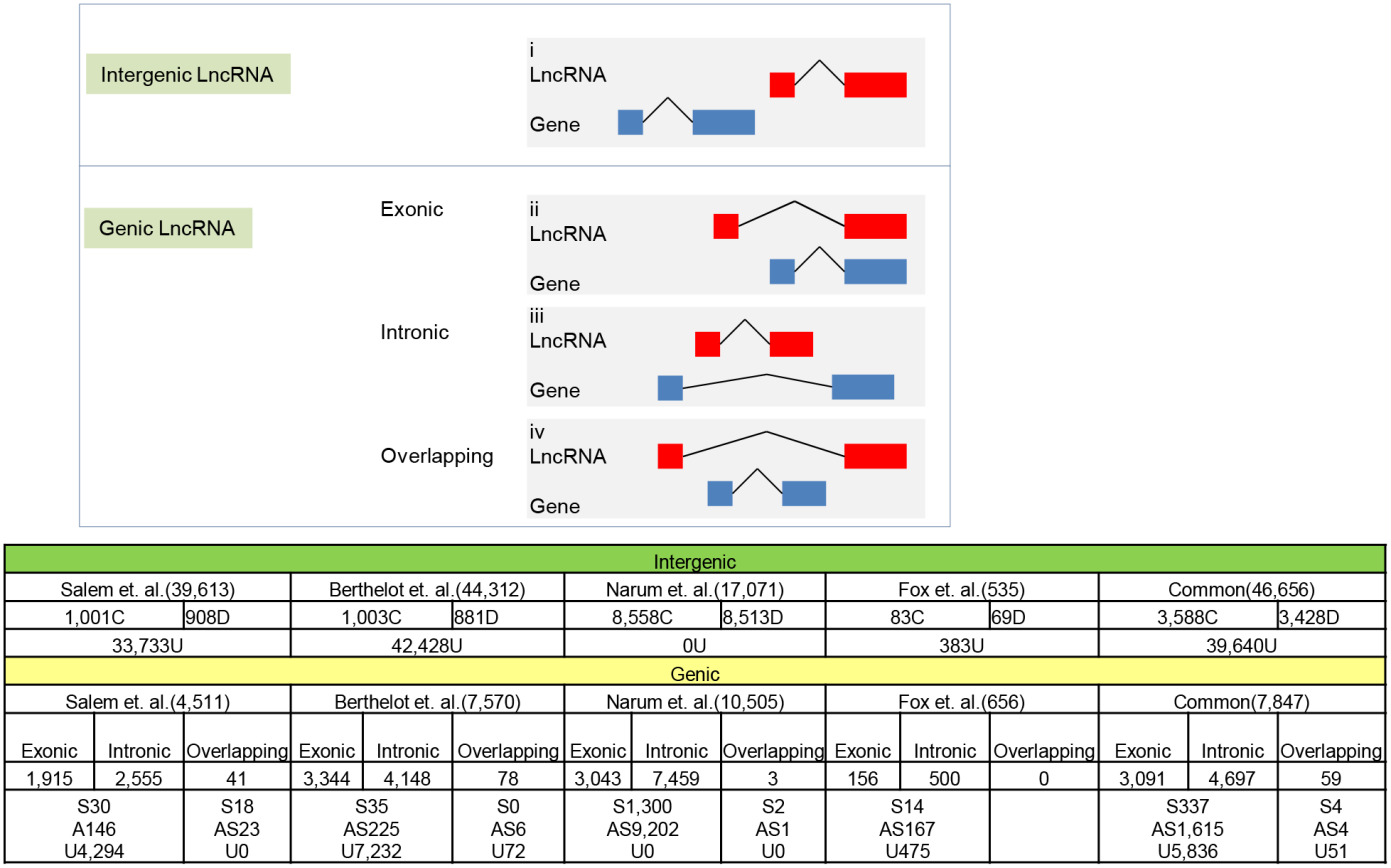
Classification of lncRNAs based on their intersection with protein-coding genes and number of lncRNAs in each class. Diagram on the top is a visual illustration of each class of lncRNAs relative to nearest protein-coding gene(s) based on genomic position and direction of transcripts. Bottom Fig in tabular format presents number of different classes of lncRNAs from each class. Numbers inside brackets following data source references indicate total number of that particular class of lncRNAs. Letters C, D, S, AS and U indicate number of convergent, divergent, sense, anti-sense and transcripts with unknown directionality, respectively.

LncRNAs are classified, based on their intersection with protein-coding genes, as genic and intergenic [9]. Some of the lncRNAs are located in transcriptionally-active regions and influence expression of neighboring genes [8, 73]. Therefore, the genomic position of lncRNAs relative to protein-coding genes can possibly provide important clues about lncRNA-mediated regulation of protein-coding genes [74]. Our data indicate that 7,847 (14.4%) of the lncRNAs intersected with protein-coding gene and thus are called genic (Fig 3). Of these lncRNAs 4,697 (8.6%), were intronic lncRNAs, existing in introns of protein-coding genes but do not intersect with any exons, and 3,091 (5.6%) exonic, sharing at least part of a protein-coding exon. Among those lncRNAs, 248 were sense and 1,488 were antisense; and 6,052 lncRNAs had an unknown orientation. In addition, there were 59 lncRNAs that completely overlapped with a protein-coding gene by containing this protein-coding gene within its intron. Fig 3 and S1 table show classification and number of lncRNAs based on their intersection with protein-coding genes. There were 46,656 (85.6%) intergenic lncRNAs in the trout genome that did not intersect but were within 15 kb of the nearest protein-coding gene. Those intergenic lncRNAs were further divided into 3,588 convergent (same sense) and 3,428 divergent (opposite sense). Consistent with our study, previous reports in humans indicate that the majority of lncRNA transcripts do not intersect with protein-coding genes [9].

### Expression of lncRNA in Different Tissues

**Fig 4:**
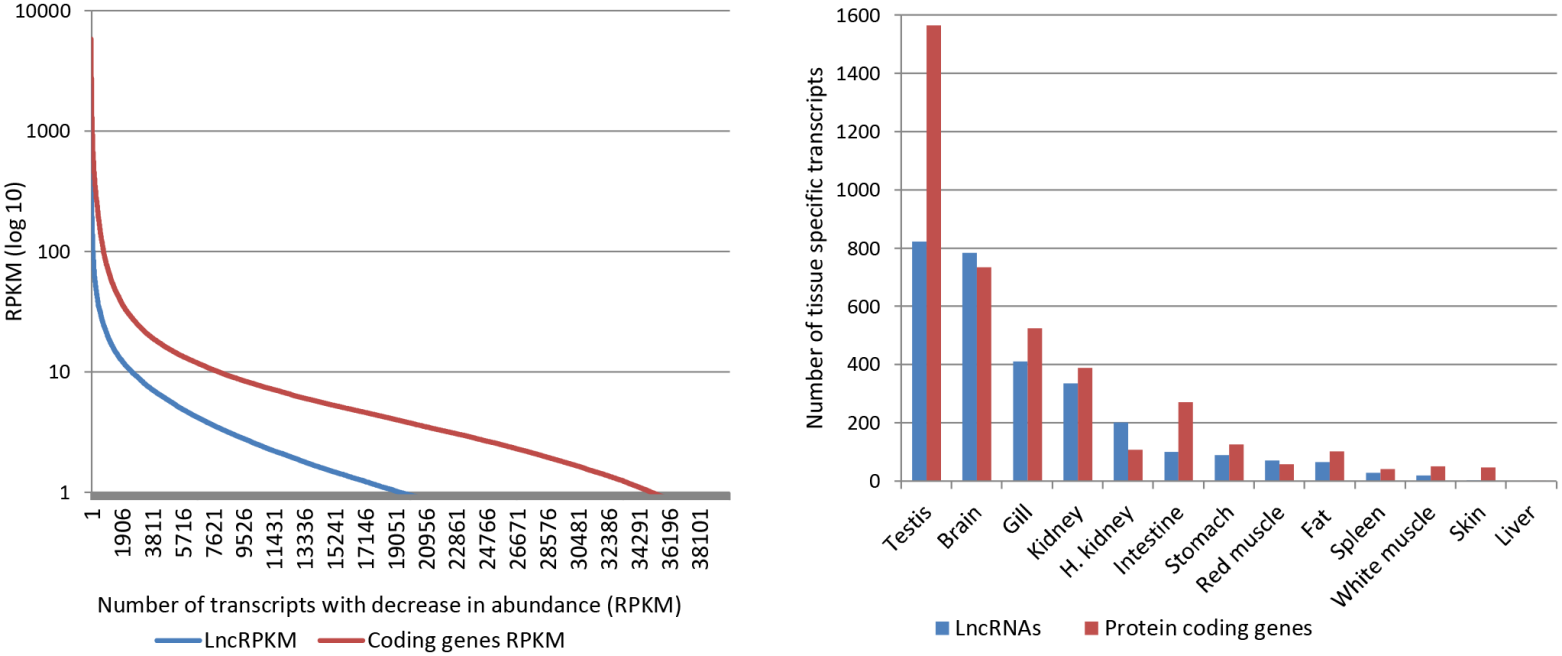
RPKM comparison of protein-coding genes and lncRNAs. Transcript abundance of lncRNAs is lower than that of protein-coding genes. Average RPKM of the most abundant 40,000 genes is 15.69 and 3.49 for protein coding genes and LncRNAs, respectively (Left). Number of tissue-specific lncRNAs and protein-coding genes in various tissues. Expression of lncRNAs and protein-coding genes showed similar patterns among different tissues (Right).

A comparison of lncRNA expression to protein-coding genes showed that transcript abundance of lncRNAs is lower than that of protein coding genes. Average RPKM (Reads Per Million per Kilo-base) of the most abundant 40,000 transcripts was 3.49 and 15.69 in LncRNAs and protein-coding genes, respectively (Fig 4). Similar trends, showing lower lncRNAs expression in all human tissues compared to mRNAs, were reported [9].

Evidence is clear that lncRNAs exhibit strict cell/tissue specificity and play a significant role in development and differentiation of tissues in plants and animals [15, 51]. Nonetheless, their tissue specificity and potential role in tissue development are not well studied in fish. Lack of sequence conservation of lncRNAs across diverse species demands study of their expression in vital tissues as a method to identify lncRNAs with tissue-specific roles in rainbow trout. In this study, lncRNA expression was studied in 13 vital tissues of rainbow trout. Out of 54,503 putative lncRNAs, 3,269 (~5.9%) exhibited expression across all tissues with a minimum RPKM value of 1.0 (S2 table). On the other hand, 2,935 tissue-specific lncRNAs (5.4%) were identified from 13 tissues (S3 table). In this report, transcripts were described as ‘tissue specific’ if their expression in one tissue was 8-fold or higher compared to the maximum value for any of the other 12 tissues with a minimum RPKM of 0.5 [43] (Fig 4). Previously, we reported 17.1% and 8.9%, respectively, for housekeeping and tissue-specific protein-coding genes [43]. To gain insight into the expression and tissue specific differences between lncRNAs and protein-coding genes, the number of each was examined in 13 different tissues (Fig 4). Testis expressed the highest number of tissue-specific lncRNAs followed by brain, gill, and kidney. Conversely, liver expressed the lowest number of tissue-specific lncRNAs followed by skin, white muscle then spleen, in increasing order. We previously reported that the number of tissue-specific protein-coding transcripts follows similar patterns in various tissues [43]. Similar to the protein-coding genes, expression patterns of tissue-specific lncRNAs can be explained in terms of tissue complexity [43].

**Fig 5:**
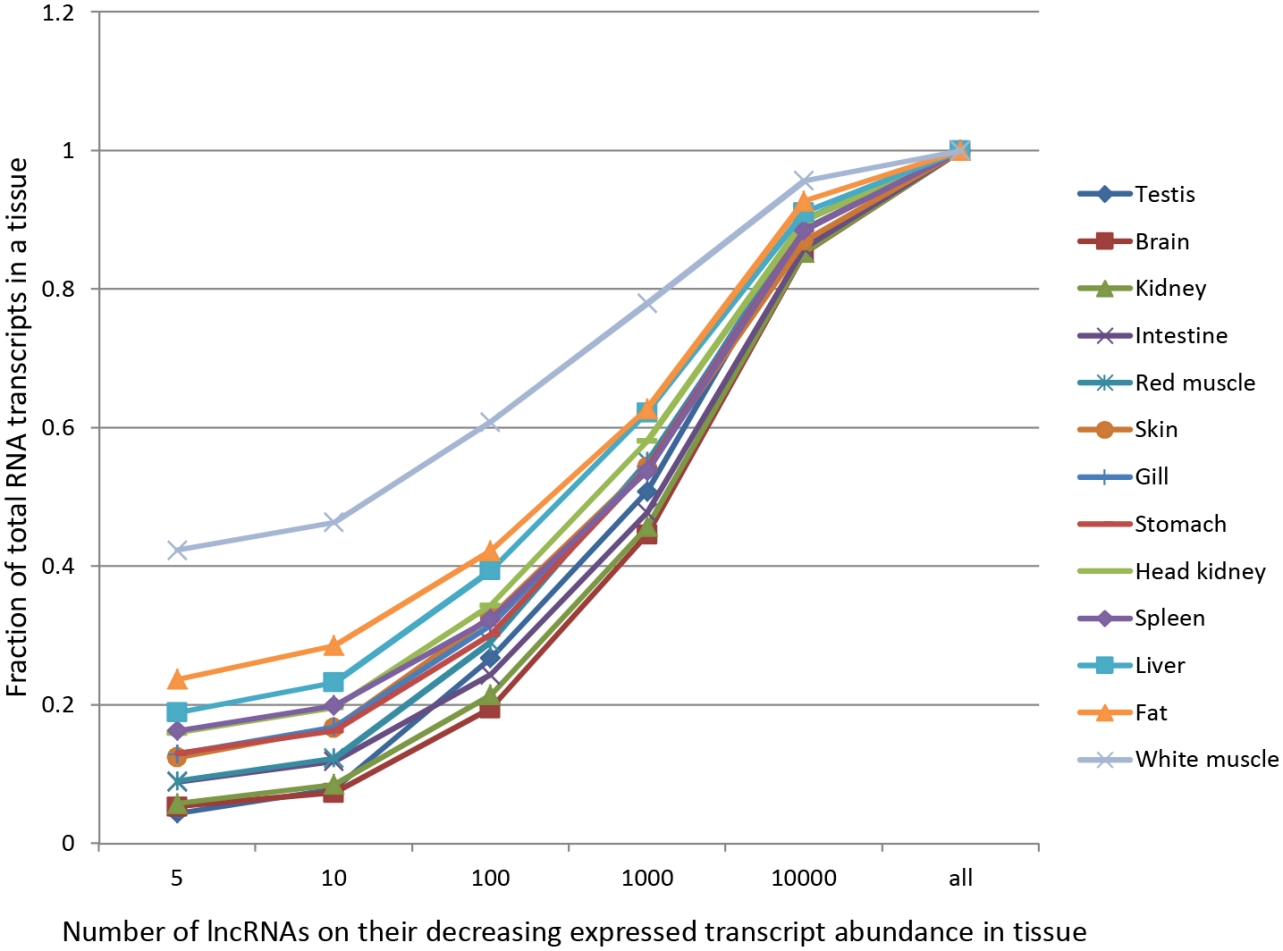
Distribution of lncRNA expression in various tissues. Proportion of the transcriptome that is contributed by the most abundant lncRNAs is plotted in various tissues. In complex tissues like brain and testis, larger number of lncRNAs were expressed with fairly equal dominance of many transcripts. On the contrary, less complex tissues like white muscle, fat and liver showed that majority of transcriptome is contributed by few dominant lncRNAs.

Previously, we showed that tissues are different in terms of the protein-coding transcriptome composition and complexity. Brain and testis possess the most complex transcriptomes. These tissues express large numbers of the genes; however, only a small part of the mRNA pool is expressed by the most abundant genes [43]. On the other hand, white muscle and stomach revealed simpler transcriptomes. These tissues express fewer genes and a greater proportion of the transcriptome comes from the most highly expressed genes. Similarly and in this study, complex tissues like brain and testis, expressed a larger number of lncRNAs with equal dominance of many transcripts (Fig 5). Conversely, white muscle, fat and liver showed less complex transcriptomes; a vast majority of the transcriptome included a few dominant lncRNAs. Similar expression patterns between protein-coding genes and lncRNAs may suggest common mechanisms of gene expression regulation and important role of lncRNAs in regulating protein-coding RNAs. Regardless, these data suggest that lncRNAs may be significant in determining tissue complexity.

### Correlation in Expression Patterns of lncRNA and Protein-Coding Genes across Tissues

Very low sequence conservation of lncRNAs hinders their molecular annotation. In order to look for possible functional significance of lncRNAs in regulating protein-coding genes, we constructed an expression-based relevance network between protein-coding genes and lncRNAs using a clustering algorithm in Multi-experiment Viewer software package (MeV) [67, 68]. In this study, biological correlation in expression patterns were compared across 13 tissues representing vastly different cellular and functional complexities. After clustering, genes of each cluster were ranked based on their entropies, and the top 20% of genes with the highest entropy were retained to construct networks. This approach identified 15 clusters containing protein-coding and lncRNA genes with strong correlation in their expression patterns (R^2^ >0.97) (S4 table). Examples of functionally important clusters include lncRNA Omy100084431 that was highly, positively correlated with splicing factor 3B (GSONMT00018324001) and transcription elongation factor SPT5 isoform X1 (GSONMT00067984001). In addition, expression of lncRNAs Omy200064145 and Omy100138726 was positively correlated with NF-kappa B inhibitor-like protein (GSONMT00082784001). Furthermore, a strong positive correlations in expression pattern between lncRNAs Omy300110093 and mitogen activated protein kinase1-like (GSONMT00053903001); Omy300072481 and thyroid hormone receptor alpha-like (GSONMT00066016001); Omy200106644 and histone deacetylase 3-like (GSONMT00058062001); and Omy300066671 and double-stranded RNA-specific adenosine deaminase (GSONMT00000999001) were observed. Proteins listed in these clusters have important functional roles in the cell including protein quality control (derlin-2) [75], RNA editing (adenosine deaminase) [76], transcriptional control (histone deacetylase 3) [77], splicing, and development. These findings nicely correlate with previously characterized molecular functions of lncRNAs in different species [23, 31, 32]. In order to explore additional underlying biological relationships between lncRNAs and protein-coding genes, more samples from different individuals and developmental stages should be studied as lncRNAs may be specific to developmental stages.

### More Stringently Selected lncRNAs

The aforementioned 54,503 putative lncRNAs were identified using filtration steps with traditional cutoff values [52, 71]. To provide an optional more stringently selected list of lncRNAs, we performed extra filtration as follows. First, we calculated the average amino acid length for the shortest 10% of the rainbow trout protein-coding genes [42]; this calculation yielded 83 amino acids. Using 83 amino acids as the cut-off value of the lncRNA, 5,836 lncRNAs were filtered out of 54,503. In addition, lncRNA containing ORF covering more 35% of its length were filtered out [66]. Second, we decreased the cut-off value for the CPC [56] from -0.5 to -1.0, which filtered out an extra 4,978 leaving 43,689 putative lncRNA. The next filtration step was performed based on location of the lncRNAs in the genome predicted from a comparison of different datasets. If any lncRNA overlapped fully or partially by more than 100 nt with another lncRNA from a different dataset, we filtered out the shortest lncRNA; this step eliminated 5,945 putative lncRNAs. In addition, we filtered out any lncRNAs that overlapped with a protein-coding gene in the sense orientation and this filtration eliminated an additional 354 lncRNAs. The last filtration step removed any single-exonic lncRNA that was within 500 nt of a protein-coding gene; as a result, 1,538 putative lncRNAs were removed. The final number of putative lncRNAs was 31,195 (S1 table). FASTA and gtf annotation files are available at http://www.animalgenome.org/repository/pub/MTSU2015.1014/. Because the criteria for distinguishing lncRNAs are still loosely defined [78], filters applied in this study (with traditional or stringent cutoff values) should be considered arbitrary, hence, the identified lncRNAs may or may not reflect biological functions. For example, some of the well characterized lncRNAs in mammals contain more than 100 AA ORF. In this study, two sets of lncRNAs were obtained with traditional or stringent cut off values. All above mentioned analyses were done using lncRNAs from the traditional filtrations.

## Supporting Information

S1 Table Number, length, exon number, and genomic classification of putative lncRNAs predicted in four transcriptomic datasets. (XLSX)

S2 Table Ubiquitously expressed lncRNAs. (XLSX)

S3 Table Tissue-specific lncRNAs. (XLSX)

S4 Table clusters containing protein-coding and lncRNA genes with strong correlation in their expression patterns. (XLSX)
